# Placenta accreta outcomes and risk factors in a referral hospital in north of Iran: A case control study

**DOI:** 10.1002/hsr2.2006

**Published:** 2024-04-10

**Authors:** Seyedeh Hajar Sharami, Forozan Milani, Sima Fallah Arzpeyma, Fereshteh Fakour, Zahra Jafarzadeh, Zahra Haghparast, Abbas Sedighinejad, Seyedeh Maryam Attari

**Affiliations:** ^1^ Department of Obstetrics & Gynecology, Reproductive Health Research Center, Al‐Zahra Hospital, School of Medicine Guilan University of Medical Sciences Rasht Iran; ^2^ Department of Radiology, School of Medicine, Poursina Hospital Guilan University of Medical Sciences Rasht Iran; ^3^ Department of Anesthesiology, Anesthesiology Research Center, Alzahra Hospital Guilan University of Medical Sciences Rasht Iran; ^4^ Department of Midwifery and Reproductive Health, Reproductive Health Research Center, Al‐Zahra Hospital Guilan University of Medical Sciences Rasht Iran

**Keywords:** outcomes, placenta accreta, risk factors

## Abstract

**Background:**

Placenta accreta syndrome (PAS) may led to heavy blood loss and maternal death. Here we analyzed the main risk factors of PAS^+^ pregnancies and its complications in a referral hospital in the north of Iran.

**Methods:**

In a case control study, all pregnant women with PAS referred to our department during 2016 till 2021 were enrolled and divided in two groups case (PAS+) and control (PAS−) based on preoperative imaging, intraoperative findings, and pathological reports. The sociodemographic features and neonatal‐maternal outcomes also were recorded.

**Results:**

The most frequent reason for cesarean (C/S) was repeated C/S (62.9%, 56/89). A significant difference showed up in the time lag between previous C/S and the present delivery (*p* < 0.001) which shows that when the time distance is longer, the risk of PAS rises (OR: 1.01 [95% CI: 1.003−1.017]). Also, a positive history of prior abortion and elective type of previous C/S were related to PAS+ pregnancies. Our other finding showed that PAS^+^ pregnancies will end in lower gestational age and have a longer duration of operation and hospitalization, heavy blood transfusion, and hysterectomy. Also, PAS^+^ pregnancies were not related to poor neonatal outcomes.

**Conclusions:**

It seems that, in addition to repeated C/S as a strong risk factor, previous abortion is a forgotten key which leads to incomplete evacuation or damage the endometrial‐myometrial layers.

## 1 INTRODUCTION

Placenta accreta syndrome (PAS) is a serious life‐threatening obstetric disorder which is characterized by placental adhesion to the uterine wall during labor.^1^ According to the depth of placental invasion into the myometrium and the adjusted organs, PAS divided to placenta accreta, increta, and percreta.^2^ Nowadays, due to the increasing rate of cesarean delivery, the incidence rate of PAS is rising worldwide.^3^, ^4^ In the same way, in a survey in Iran in 2018, the rate of cesarean sections was rising and reported around 50%–60% of total deliveries.^5^ Also, in another study in Iran on 2021, Kasraeian et al.^6^ revealed a rate of 1/263 cases for PAS^+^ pregnancies which were one of the most important high reported prevalence of PAS.

PAS is usually diagnosed using sonography with 87% sensitivity and 98% specificity.^7^ Other diagnostic modalities are magnetic resonance imaging (MRI) and histopathological evaluation.^8^ The most important complications of PAS are massive hemorrhage, maternal death, hysterectomy, ICU admission, and prolong hospitalization.^3^, ^4^, ^5^, ^6^, ^7^, ^8^ In PAS^+^ pregnancies, due to the high amount of blood loss, it might be necessary to change the treatment plan to cesarean‐hysterectomy (a C/S followed by the hysterectomy).^8^ Obstetricians prefer to terminate such pregnancies earlier in tertiary centers, and in some cases, it requires several surgical specialists in the surgery setting and it also may need to massive blood transfusion.^7^, ^8^

Despite the strong relationship between previous C/S and PAS, all of repeated C/S are not involved with, and on the other hand, there are some reports of primiparus pregnancies complicated with PAS.^9^ So here we studied the other risk factors of PAS^+^ and the maternal and neonatal outcomes.

## 2 MATERIALS AND METHODS

### 2.1 Study design

This is a case control study from March 2016 to 2021.

### 2.2 Study population

All the pregnant women with a prior cesarean section (PAS− and PAS+ confirmed with preoperative imaging, intraoperative findings, and histopathological reports) referred to obstetrics and gynecology center at Rash Hospital.

### 2.3 Sample size and sampling methods

Sample size was calculated through the following formula and the data of Farquhar et al.^8^ with a confidence interval of 95%, a margin of error of 0.05, and 80% power of the study.

$$\begin{array}{l} \cdots \\ n=\frac{\left[z_{1-\frac{a}{2}}\sqrt{rp(1-p)}+z_{1-\beta }\sqrt{p_{case}(1-p_{case})+p_{control}(1-p_{control})}\right]2}{{\left(p_{case-}p_{control}\right)}^{2}} \end{array}$$

Sampling done using convenient sampling methods.

Inclusion criteria includes preoperative ultrasonographic or MRI evidence of PAS, intraoperative findings, postoperative pathological sample report with loss of the layer interfaces between the placenta decidua and myometrium (Nitabuch layer). Patients with no ultrasonography of the placenta excluded from the survey.

### 2.4 Study groups

**Figure 1:**
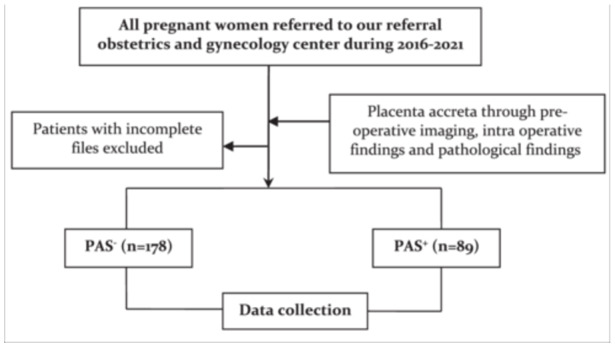
Flowchart. PAS, placenta accreta syndrome.

The case group (PAS+) consisted of all pregnant women placenta accrete syndrome and the control group (PAS−) was consisted of pregnant women without PAS. Both the groups were matched for maternal age, parity, and gestational age (Figure 1). Using a data collection sheet, all variables of the study were recorded. The checklist contained of two main sections: The first section: sociodemographic characteristics; the second section: maternal and neonatal outcomes.

#### 2.4.1 Sociodemographic features

Age, menarche age, parity, past medical history (diabetes, hypertension), ART, number of previous C/S (1−n), body mass index, features of the prior cesarean section (gestational age, elective/emergency), the time lag between previous C/S and the present delivery, the main reason of previous cesarean (fetal distress/breech presentation/arrest of labor in the active phase/placenta previa/repeat cesarean delivery), postpartum complications (ICU admission/infection/hemorrhage), history of dilation and curettage or myomectomy, previous IUD implantation, and sexual transmitted disease including Herpes, trichomonas, wart, gonorrhea.

#### 2.4.2 Maternal and neonatal outcomes

Sonography or pathology report on the severity of invasion (accreta/percreta/inccreta), gestational age, duration of hospitalization, operating time, the blood loss, postoperative hemorrhage, thromboembolic events (DVT, DIC, PE), bladder injury, ureter injury, transfusion of blood products (packed cell, FFp, platete), acute kidney injury, ICU admission, maternal death (during 40 days after delivery), IUFD, APGAR score, NICU admission, and neonatal death (during 42 days after delivery).

#### 2.4.3 Data analysis

The findings were statistically analyzed using SPSS software v. 22.0 (SPSS Inc.). The frequency and percentage were used to report the qualitative results. For the quantitative data, mean and standard deviation were used. To comparison the variables, independent *T*‐test, *χ* ^2^, and fisher exact test were used. Mann–Whitney test and Wilcoxon test used in nonparametric results. *p* < 0.05 is considered statistically significant.

## 3 RESULTS

A total number of 267 pregnant women with previous cesarean were evaluatedand 89 pregnant women with PAS^+^ were allocated in the case group and 178 women without PAS (PAS^–^) allocated in the control group.

The age ranged from 19 to 44 years with a mean age of 32.8 ± 4.8 years in the case group and 26.9 ± 2.9 years in the control group (*p* > 0.05). The distribution of demographic characteristics in the two study groups is shown in Table 1. Sociodemographic variables are comparable between study groups except for history of abortion (Table 1).

**Table 1:** Comparison of sociodemographic features between the case and control groups conducted Rasht Hospital, Iran, 2016–2021.

	Case (%) *n* = 89	Control (%) *n* = 178	*p* Value
Age (mean ± SD) (year)	32.8 ± 4.8	26.9 ± 2.9	0.414
The mean gestational age at the time of delivery (weeks)	35.1 ± 2.9	37.7 ± 1.1	0.001*
Body mass index (mean ± SD) (kg/m^2^)	26.9 ± 2.9	26.7 ± 2.9	0.084
Age at menarche (mean ± SD) (year)	11.3 ± 1	10.8 ± 2.5	0.069
Previous abortion (*n*)	37 (41.6)	36 (20.2)	0.001*
Parity (*n*)			
Nulliparous	48 (53.9)	99 (55.6)	0.534
Multiparous	41 (46.0)	79 (44.8)	
Sexual transmitted disease (STD)			
Herpes	1 (1.1)	6 (3.3)	0.358
Wart	6 (6.7)	11 (6.1)	
Past medical history			
Diabetes	37 (41.5)	86 (48.3)	0.474
Hypertensive disorders	29 (32)	54 (30.3)	
History of dilation and curettage (*n*)	13 (14.6)	21 (11.8)	0.931
History of myomectomy (*n*)	8 (8.9)	19 (10.6)	0.340
Previous IUD implantation (*n*)	2 (2.4)	9 (5.0)	0.654

* *p* < 0.05.

**Figure 2:**
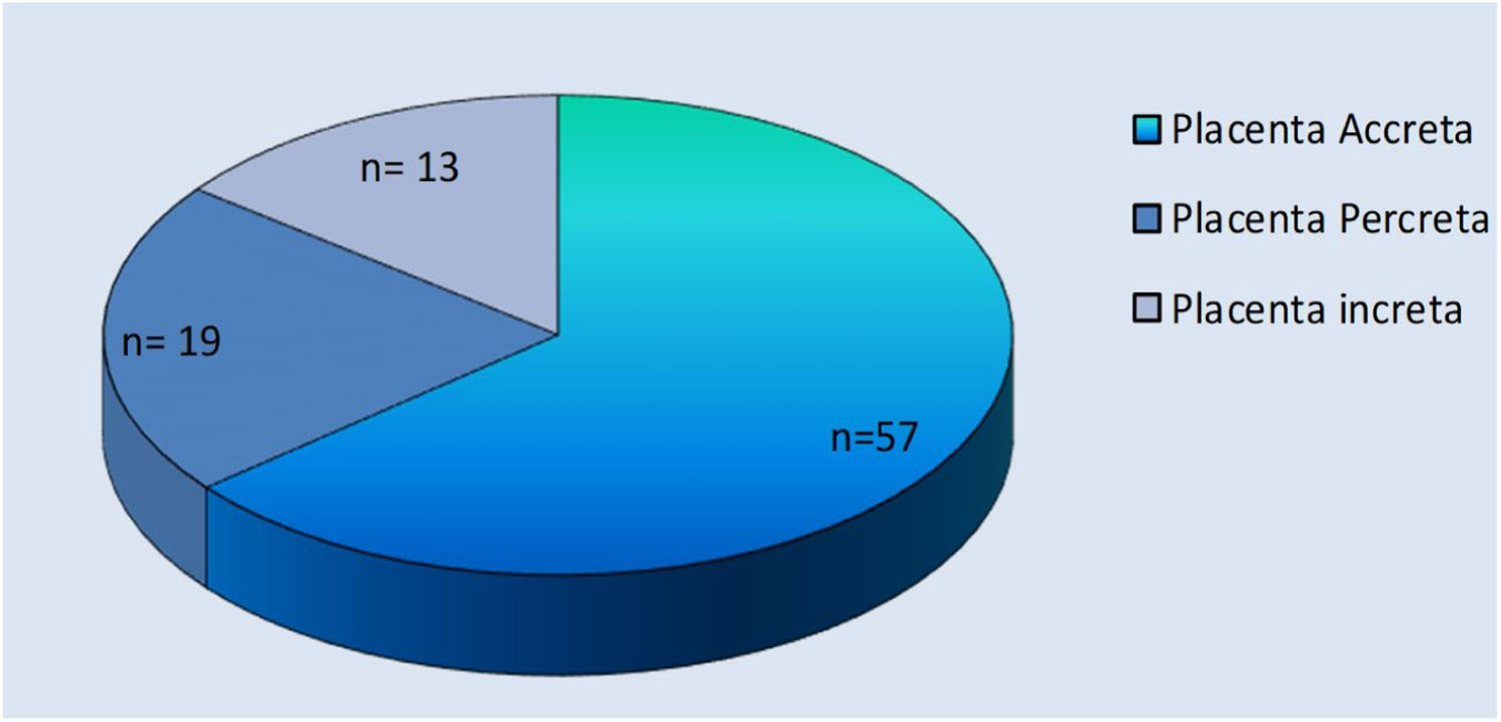
Different types of PAS (based on pathology report. PAS, placenta accreta syndrome.

In the case group, postoperative diagnosis (pathology) was placenta accreta in (64%, 57/89), placenta percreta (21.3%, 19/89), and placenta increta (14.6%, 13/89) (Figure 2).

The most frequent reason for cesarean delivery was repeated C/S in both the study groups.

The time lag between previous C/S and the present delivery is significantly longer in the case group as compared to control group (*p* < 0.001). The details are shown in Table 2.

**Table 2:** Comparison of prior cesarean section features between case and control groups conducted at Rasht Hospital, Iran, 2016–2021.

Prior cesarean features	Case (%) *n* = 89	Control (%) *n* = 178	*p* Value
Number (mean ± SD)	1.2 ± 0.7	1.3 ± 0.5	0.486
gestational age (weeks)	36.4 ± 2.9	37.8 ± 1.7	0.590
Feature			
Emergency	29 (32.6%)	116 (65.2%)	0.001*
Elective	53 (59.5%)	62 (34.8%)	
Main reason			
Fetal distress	10 (11.2%)	40 (22.4%)	0.621
Breech presentation	9 (10.1%)	17 (9.6%)	0.624
Arrest of labor	8 (9%)	42 (23.6%)	NS
Placenta previa	6 (6.7%)	‐‐‐‐	0.001*
Repeat C/S	56 (62.9%)	79 (44.4%)	0.373
The time lag between previous C/S and the present delivery (month)	63.2 ± 39.6	37.2 ± 48	0.001*
Postpartum complications			
ICU admission	11 (12.3%)	17 (9.5%)	0.387
Infection	3 (3.3%)	5 (2.8%)	0.476
Hemorrhage	6 (6.7%)	11 (6.2%)	0.870

* *p* < 0.05.

The mean gestational age at the time of delivery was 35.1 ± 2.9 and 37.7 ± 1.1 weeks in the case and control groups, respectively (*p* < 0.001). It shows that in PAS^+^ pregnancies, the time of delivery is earlier. The average duration of hospitalization was 3.5 ± 1.9 days in the case group which was longer than the control group (*p* < 0.001). In the same way, operation time was longer in the case group (74.42 ± 29.4 vs. 55.08 ± 13.09 min) (*p* < 0.001). The mean blood loss in the case group was 2544 mL which was significantly more than the control group (*p* < 0.001). Blood transfusion happen in 53.93% of case and 21.9% of control patients (*p* < 0.001). Cesarean hysterectomy performed in 39 mothers in the case group and only one mother in control group (*p* < 0.001). Furthermore, ICU admission was in all patients in the case group and only 6.17% of the control group (*p* < 0.001). Neonatal characteristics did not differ between the two study groups (Tables 2 and 3). The locations of the PAS within the uterine cavity are based on the preoperative imaging studies or preoperative findings are shown in Table 4.

**Table 3:** Comparison of neonatal and maternal outcomes between the two study groups.

	Case (%) *n* = 89	Control (%) *n* = 178	*p* Value
The average duration of hospitalization (days)	3.5 ± 1.9	2.3 ± 1.2	0.001*
The operation time (min)	74.4 ± 29.4	55.1 ± 13.1	0.001*
The mean blood loss (mL)	2544	1750	0.001*
Blood transfusion (%)	53.93	21.9	0.001*
Cesarean hysterectomy	39	1	0.001*
ICU admission	89 (100%)	6.17%	0.001*
IUFD	1 (1.1%)	2 (1.1%)	0.766
NICU admission	18 (20.2%)	26 (14.6%)	0.640
APGAR score 1^ST^ min			
≥7	12 (13.5%)	5 (2.8%)	0.458
<7	77 (86.5%)	173 (97.2%)	
APGAR score 5^ST^ min			
≥7	2 (2.5%)	3 (0.6%)	0.368
<7	87 (97.5%)	175 (99.4%)	

* *p* < 0.05.

**Table 4:** The locations of the PAS within the uterine cavity base on the preoperative imaging studies or preoperative findings.

PAS location	*n* (%)
In the lower uterine segment	38 (42.7%)
On anterior uterine wall	51 (57.3%)
On the posterior uterine wall	0 (0%)
On fundal region of the uterine cavity.	0 (0%)

Abbreviation: PAS, placenta accreta syndrome.

Table 5 shows multivariate logistic regression on the different risk factors associated with PAS+ pregnancies. The analysis shows a significant relationship between PAS+ pregnancies and a history of prior abortion and type of cesarean section (Table 5).

**Table 5:** Multivariate logistic regression table shows factors associated with PAS+, Rasht Hospital, Iran, 2016–2021.

Characteristics	*B*	SE	Wald	OR (95% CI)	*p* Value
Age	0.008	0.043	0.030	1.008 (0.926−1.096)	0.862
Gestational age	0.423	0.304	1.942	1.527 (0.842−2.770)	0.163
Parity	−0.612	0.397	2.368	0.542 (0.249−1.182)	0.124
Age at menarchae	−0.107	0.205	0.275	0.898 (0.601−1.342)	0.600
History of abortion	1.142	0.466	6.003	3.133 (1.257−7.811)	*0.014
STD infection	−1.748	1.067	2.687	0.174 (0.022−1.408)	0.101
Time lag between previous C/S and now (month)	0.009	0.005	3.018	1.009 (0.999−1.018)	0.082
Types of cesarean section	−1.788	0.522	11.722	0.167 (0.060−0.466)	*0.001

Abbreviation: PAS, placenta accreta syndrome.

* *p* < 0.05.

## 4 DISCUSSION

PAS is a life threatening situation which leads to severe maternal morbidities and mortalities specially due to the high amount of blood loss. Of course, early diagnosis can be a golden key to save such mother's life and in pregnancies with more risk factors of PAS, such as placenta previa and scarred uterus, it should be considered as soon as possible. Our results showed the significance of PAS^+^ pregnancies management due to the probable morbidities such as heavy blood loss, ICU admission, prolonged hospitalization, and permanent infertility due to hysterectomy.

The most famous hypothesis for the PAS etiology is the endometrial‐myometrial interface defect at the previous C/S scar or any other incisions the endometrium which results in abnormal decidualization and deep anchoring of chorionic villi and trophoblast infiltration which can invade the myometriuma and serosa layer.

A fertilized egg needs an environment of both oxygen and collagen for implantation. Because the uterine scar possesses the both, during a repested pregnancy, embryos will be implanted at the incision site.

Also, there are several parts of discontinuity and thinning in a repeated C/S uterine results in a poor growth of the tunica intima and a weaken myometrium.

In fact, the scar of hysterotomy may damage the terminal layer on the implantation site, and therefore, in the next pregnancies placenta will be directly in touch with myometrium.^10^, ^11^

In the same way, our results showed that all patients had a positive history of previous C/S, but, there were no relationship between the number of previous C/S and the risk of PAS. Despite our results, in another study on evaluation of PAS during 4 decades on 2013, the only variable which differ statistically significant was the number of previous C/S.^12^ Along with our results, Erfani et al.^10^ showed a higher rate of previous C/S in PAS^+^ pregnancies (89.9% vs. 46.8%; *p* < 0.001). We found no relationship between maternal age and the risk of PAS, maybe due to the matching process in include patients in the control group. In another case control study, Farquhar et al.^8^ showed that in pregnant women older than 40 years old, the risk of PAS is higher than those younger than 30 years old. Also, Meng et al.^13^ showed that with rising in maternal age, the risk of PAS rises with no relationship to the history of C/S.

Among other demographic features, there were a significant relationship between previous elective C/S, the long inter pregnancies interval, positive history of placenta previa and a history of abortion. Similar to our results, there are several studies which declare that placenta previa is an independent risk factor for placenta accrete most likely due to the overlap of their common risk factors and underlying causes.^11^, ^12^, ^14^ Also, due to the hematoma or remnant placental tissue within the myometrium layer, a positive history of abortion may be a risk factor of a weak point for future PAS.^15^

There are some studies declared that with a short interval between the previous C/S and the present pregnancy, the risk of PAS rises^16^, ^17^ which may be related to the incomplete healing of the scar of the previous incision. But we found the opposite, and in short term inter pregnancies interval, PAS happened more frequent. Abdelrahman et al.^18^ declared that despite the overall belief, there is no relationship between the time lag between the two C/S and PAS.

Our other findings showed the more frequent type of previous C/S was elective surgery. It has been discussed that on emergent C/S which mostly happens during labor phase, proper dilation of lower segment presents a thin uterine wall and a low site incision which leads to a good prognosis for no PAS in future. On the other hand, in elective C/S, lower segment has a thick myometrium and healing with fibrosis will led to a defect named “Niche” which has a high risk of PAS in implantation of ovum on this sit. On the other hand, when a uterine is on delivery phase, it is immunologically active and will led to a stronger healing process, and in non‐immunological environments, the risk of PAS may be higher.^10^, ^11^, ^12^, ^13^

Our study showed that PAS^+^ pregnancies will end in lower gestational age, longer duration of operation and hospitalization, heavy blood transfusion, and hysterectomy. Along with our results, Shi et al.^14^ showed that elective C/S has higher rate in PAS+ pregnancies with lower gestational age. Farquhar et al.^8^ showed that PAS^+^ pregnancies had more positive history of C/S and resulted in ICU admission and hysterectomy. Erfani et al.^10^ resulted a higher amount of blood loss and transfusion in PAS^+^ pregnancies. In a 5‐years survey, Kasraeian et al.^6^ resulted that the mean amount of blood loss during PAS + C/S was 2.5 L and the average amount of blood transfusion was two packed cell. About the neonatal results, there were no significant relationship between PAS^+^ pregnancies and poor neonatal outcomes.

## 5 CONCLUSION

Accreta syndrome is a life threatening situation which may led to heavy blood loss and maternal death. Our findings showed that in addition to repeated C/S as a strong risk factor, previous abortion is a forgotten key which may become underestimated as a major clue. In our country, due to the culture and religious beliefs, abortion is not legal, so, there are a high number of illegal abortion which leads to incomplete evacuation or damage the endometrial‐myometrial layers. It seems that government should consider novel solutions to decrease such abortions. The other important risk factor in our survey was elective C/S which has a high rate and must be decreased through to encourage primigravide mothers for normal vaginal delivery and avoid of C/S with no medical criteria.

## AUTHOR CONTRIBUTIONS

**Seyedeh Hajar Sharami**: Conceptualization; methodology; writing—review and editing. **Forozan Milani**: Conceptualization; methodology; writing—review and editing. **Sima Fallah Arzpeyma**: Conceptualization; data curation; methodology; visualization; writing—review and editing. **Fereshteh Fakour**: Validation; writing—original draft. **Zahra Jafarzadeh**: Data curation; writing—original draft. **Zahra Haghparast**: Data curation; writing—original draft. **Abbas Sedighinejad**: Writing—review and editing. **Seyedeh Maryam Attari**: Project administration; supervision; writing—review and editing.

## CONFLICT OF INTEREST STATEMENT

The authors declare no conflict of interest.

## ETHICS STATEMENT

The ethics committee of Guilan University of Medical Sciences approved the survey (IR.GUMS.REC.1399.664). All cases filled the written informed consent before the study begins. Informed consent was taken from all participants.

## TRANSPARENCY STATEMENT

The lead author Forozan Milani affirms that this manuscript is an honest, accurate, and transparent account of the study being reported; that no important aspects of the study have been omitted; and that any discrepancies from the study as planned (and, if relevant, registered) have been explained.

## Data Availability

Related data of this project are available on request. Data Availability statement supporting data are available in Reproductive Health Research Center, Al‐Zahra Hospital, School of Medicine, Guilan University of Medical Sciences, Rasht, IRAN.
